# Platform pregnancy and digital birth: social (de)construction of bodies and online discourses on three-dimensional ultrasound images in the transition to parenthood

**DOI:** 10.3389/fsoc.2026.1721516

**Published:** 2026-06-10

**Authors:** Ilenia Picardi, Maria Carmela Agodi

**Affiliations:** Department of Political Science, University of Naples Federico II, Naples, Italy

**Keywords:** 3D/4D ultrasound imaging, digital foetus, parental wellbeing, platform pregnancy, transition to parenthood

## Abstract

Three-dimensional (3D)and four-dimensional (4D) ultrasound technologies have evolved from their clinical applications to become integral components of in digital platforms, private commercial settings, and online communities. This article explores how online discourses pertaining to 3D and 4D foetal ultrasound images contribute to the reconfiguration of parental wellbeing during the transition to parenthood. The study draws upon feminist science and technology studies and digital sociology in order to demonstrate how pregnancy, foetal visibility, and parental subjectivities are increasingly mediated by digital infrastructures, and to conceptualises the practices of transition to parenthood as part of a broader process of “platform pregnancy” and “digital birth”. The article employs a Multimodal Critical Discourse Analysis to empirically study the content of posts and images shared on two UK-based parenting forums. The analysis focuses on how parents — predominantly expectant mothers — describe, interpret, and emotionally negotiate their encounters with 3D/4D ultrasound scans, and how these experiences are collectively discussed and reframed within online environments. The findings demonstrate that 3D/4D ultrasound images exhibit an ambivalent role in parental wellbeing. Whilst often anticipated as sources of reassurance, fostering bonds, and facilitating emotional connections with the foetus, these experiences can also generate feelings of anxiety, discomfort, and frustration, particularly when the actual experience of viewing diverges from culturally idealised representations of the foetus and the concept of ideal parenthood. Online forums have been shown to function as affective infrastructures in which these tensions are negotiated through storytelling, peer support, humour, and the circulation of images. This enables the collective management of uncertainty and emotional distress. The article presents a conceptual argument that the circulation of ultrasound images on digital platforms contributes to the emergence of the “digital foetus”, defined as a hybrid entity produced through the entanglement of medical imaging, parental affect, and platform-mediated visibility. This process anticipates social recognition prior to biological birth, thereby giving rise to what is termed “digital birth”. The article employs the concept of “digital timescapes”, the article to demonstrate how digital technologies compress, accelerate, and reorganise the temporalities of pregnancy, thereby reshaping the rhythms of anticipation, waiting, and becoming a parent. The article contributes to sociological debates on reproduction, embodiment and parenthood in the digital age by foregrounding the interplay between digital technologies, visual culture and wellbeing.

## 1 Introduction

The study of parenthood provides a privileged window through which to observe social transformation. The process of becoming a parent is a profoundly social process, shaped by a variety of factors including demographic trends, welfare systems, gender relations, values, norms and cultural imaginaries. The transition to parenthood reveals the intertwining of intimate experiences and diverse life trajectories with broader institutional, political, and technological contexts. Scientific and medical institutions, in conjunction with markets and digital infrastructures, are assuming an increasingly pivotal role in these transformations, particularly within most European and North American countries, where reproductive possibilities, health practices, and daily parental experiences have been deeply reshaped by biomedical and digital technologies.

Assisted reproductive technologies (ART), oocyte cryopreservation, genetic testing, and surrogacy have expanded the range of reproductive possibilities, thereby challenging previous limitations related to age, biology, or social status. On the other hand, diagnostic technologies, including ultrasound, in-utero magnetic resonance imaging (iuMRI), and the more recent three- and four-dimensional (3D/4D) imaging techniques, have provided new tools for the monitoring of the maternal and foetuses heath. At the same time, these technologies have transformed the relationship between pregnant women, foetuses, medical professionals, and broader publics. Digital platforms, apps, and online communities have further amplified these processes, generating new modes of sharing, interpreting, and negotiating reproductive experiences.

Recent research has expanded the understanding of how reproduction is shaped by technoscience, markets, and governance investigating the role of biotechnologies in reconfiguring the dimensions of parenthood (Valdez, 2022; Schurr, 2017; Salter, 2022; Jasanoff, 2005), and analysing the intertwining between digital health technologies and parental wellbeing (Bäckström et al., 2022). This paper, inscribed in the theoretical framework offered by the Science and Technologies Studies (STS), contributes to this field of research by investigating the intersections between medical technologies, markets, and digital infrastructures in shaping contemporary parenthood. The focal point of this study is on the interplay between two practices that have gained significant traction in the field of obstetrics: three-dimensional foetal ultrasound and the dissemination of ultrasound images through digital platforms.

The advent of three-dimensional and four-dimensional ultrasound technologies has precipitated a technological evolution of routine two-dimensional clinical scans. These technologies differ in their technical features, as well as their diagnostic and social meanings. Three-dimensional and four-dimensional scans generate volumetric images of the foetus, with four-dimensional scans being capable of real-time movement. Conventional two-dimensional ultrasound is predominantly utilised for diagnostic and screening purposes within public healthcare systems. However, the emphasis on visualisation and recognisability of three- dimensional and four-dimensional technologies is such that they are considered to surpass that of clinical measurement. The utilisation of these practices within routine obstetric care is limited, with their prevalence being established through their provision by private, commercial clinics as a distinctive experience offered to those pregnant women wishing to partake in such an activity.

The article specifically examines how online narratives of 3D/4D ultrasound practices, when embedded in online parental communities, contribute to the social construction of the “digital foetus” (Agodi et al., 2023) and reshape parental well-being. It is argued that ultrasound technologies operate not merely as diagnostic devices but as sociotechnical assemblages, wherein medical, cultural, emotional, and economic dimensions converge. The study analyses narratives and exchanges on parenting forumsin order to explore how such practices generate ambivalent effects, including joy, reassurance, and bonding, but also anxiety, regret, and aesthetic concerns.

The concepts of “digital birth, and “platform pregnancy” are proposed as analytical tools to capture how digital visualisation practices anticipate and reshape the transition to parenthood, reorganising affective investments and parental identities before birth. This conceptual framework enables us to examine how the intersection of medical, digital, and visual technologies is reconfiguring the timeline of parenthood. The study investigates the effects of the digitally mediated anticipation and compression of pivotal experiential moments of pregnancy on parental well-being and the embodied process of making sense of experiences. By doing so, we aim to contribute to sociological studies on parenthood and stimulate a wider public debate on parental wellbeing in the digital age.

The article is structured as follows. The next section outlines the theoretical framework, drawing on feminist studies and science and technology studies the concept of *public foetus* (Duden, 1994), *corporealization* (Haraway, 1997)*, apparatuses* (Barad, 2007) to analyse the online narrative surrounding three- and four-dimensional ultrasound technologies as practices of transition to parenthood. A methodological note is following, on the analysis of online narratives and forum discussions. The empirical sections explore how 3D and 4D ultrasound images circulate within parental digital communities and how these practices are reshaping emotional experiences of pregnancy. The final section discusses the notions of “digital birth” and reflects on the implications of platform pregnancy for the reconfiguration of parenthood’s temporalities in the digital age.

## 2 From imagining to corporealizing the Foetus

Within the social sciences, the analysis of foetal images has been the subject of numerous and contrasting reflections, focusing on how this technology has transformed the lived experiences of pregnancy and parenthood, but also on the social and political meanings that foetuses have acquired once the human body—and in particular, the maternal womb—became “transparent” (Van Dijck, 2005).

Originally developed as a diagnostic tool in the 1930s–40s, by the 1970s, ultrasound had become an integral part of both prenatal care routines and the ritualization of pregnancy in Western societies (Clement et al., 1998; Van Dijck, 2005). Thanks to sonographic technology, foetal bodies, which previously may have been only imagined (Black, 1992), became objects of public visibility. As Petchesky (1987: p. 69) argues, ultrasonography “rendered the once opaque womb transparent, stripping the veil of mystery from the dark inner sanctum, and letting the light of scientific observation fall on the shy and secretive foetus.”

Feminist and STS scholarship have been crucial in interrogating the ambivalent consequences of these transformations. Beginning with the early analyses of ultrasound imaging as a tool that rendered the foetus a *public object* (Duden, 1994) to more recent analyses, introducing the concept of “digital foetus” (Agodi et al., 2023), scholars have highlighted how visual and digital technologies of reproduction simultaneously have expanded possibilities for bonding and knowledge, while also produced new forms of anxiety, surveillance, and control. Pioneering works such as those of Petchesky (1987) and Duden (1994) emphasised how the visualisation of the foetus through sonography detached it symbolically from the maternal body, rendering it a “public foetus” and, at times, obscuring the presence of the woman herself. These studies showed how, while on the one hand ultrasound has become a crucial medical tool for monitoring pregnant women’s health, on the other hand it contributed to the medicalization of pregnancy (Oakley, 1984; Roberts, 2012), to the erasure of pregnant women in favour of foetal centrality (Petchesky, 1987; Stabile, 1994), and to the construction of the foetus as an autonomous patient or citizen (Franklin, 1991; Casper, 1998). In this sense, ultrasound fuelled an epistemic shift in pregnancy knowledge, displacing women’s embodied knowledge in favour of the technological visibility of the foetus (Sandelowski, 1994).

This reconfiguration reshaped maternal perception—transforming pregnancy from an embodied sensation to an image-mediated relation—and had a profound impact on public debates about reproduction. Foetal images became powerful rhetorical tools in controversies surrounding abortion, often mobilised to support discourses of foetal personhood and normative ideals of motherhood. The “autonomized foetus” emerged as a cultural and political object: disseminated in the media, used in advertising (Taylor, 2008), and mobilised in pro-life campaigns (Petchesky, 1987; Lupton, 1994). In accordance with this feminist perspective, and focusing her reflection on the agency of technology in pregnancy and the construction of bodies, Haraway (1997) defined the foetus, as it was made visible by technology, as a cyborg: a hybrid organic-technological entity, “free-floating” and hyper-visible on screens, which condenses struggles over family, nation, choice and the future. Haraway identifies the foetus as a multiple “work object,” a performative icon that is generated by different social worlds (mothers, fathers, doctors and politicians).

Notwithstanding the tensions that have characterised the public discourses surrounding foetal imageries, ultrasound technology rapidly consolidated in national health services across Western countries, with protocols varying by context. Its success cannot be explained solely in diagnostic terms but also by its ability to respond to parental anxieties, encouraged by healthcare professionals—and increasingly by markets—as a practice of reassurance and bonding.

Research has investigated how imaging technologies mediate parental perceptions and identities. Foetal images assist parents in decision-making, acquiring emotive as well as practical value. The “parental gaze,” combined with medical commentary, contributes to transforming both foetal and parental identities even under conditions of uncertainty (Lie et al., 2019). Ultrasound sessions also enhance fathers’ experiences, functioning as a prosthetic device that grants access to a realm traditionally mediated through women’s embodied knowledge (Sandelowski, 1994). They have become central events in fathers’ pregnancy experience, intensifying the reality of the foetus and reinforcing the primacy of vision as a means of knowing the unborn (Draper, 2002; Becker and Hann, 2021).

The reflections presented so far support the widespread idea among sociology scholars that recognised the routine obstetric ultrasound as a “hybrid practice” (Taylor, 2008), simultaneously diagnostic and social, medical and cultural. Ultrasound is not only a clinical examination but also a social event that reinforces the centrality of vision in Western epistemologies of the body. It provides biomedical information about foetal development, but also generates affective experiences, social narratives, and symbolic constructions that shape how individuals and couples experience becoming parents. The boundaries between clinical and social components are shifting and permeable (Mitchell, 2001; Van Dijck, 2005), often producing tensions: clinical aims intrude upon the social pleasures of scanning, while social expectations disrupt clinical objectives. Sonograms, in this sense, are semiotic objects with multiple and shifting meanings, historically and culturally specific (Mitchell and Georges, 1997).

Introducing the concept of “digital foetus” (Agodi et al., 2023), we investigated “foetal corporealization” occurred through the combined actions of medical and digital “apparatuses.” Haraway’s (1996) notion of *corporealization* is specifically handy: bodies are not pre-given but materialised through practices involving both human and non-human actors. Our study focuses on how foetal corporealization emerges through the hybridization of sonographic techniques and digital cultures, in which actions and narratives by professionals and parents, around these digital images, shape foetal bodies and re-signify them. Barad’s (2007) agential realism further support this perspective by conceptualising technologies as “material-discursive practices” in which apparatuses do not passively capture reality but actively produce phenomena. According to Barad, apparatuses enact “agential cuts” that make certain aspects of reality visible, while excluding others. Applied to reproductive imaging, this means the foetus is not simply “revealed” by ultrasound but materialised through intra-actions among biological processes, medical technologies, digital platforms, and discursive practices. The ultrasound machine, rendering software, parental gaze, and online forums all operate, together with the scanned living matter, as interconnected apparatuses. Through these intra-actions, the foetus becomes not only a clinical object but also a socio-cultural figure—a digital entity that shapes parental emotions and identities.

From this Baradian perspective, digital foetal images are not inert representations but active agents in the configuration of parenthood. They can comfort by providing reassurance but also generate distress when aesthetic or medical interpretations circulate online. They co-constitute the “digital foetus,” a hybrid figure shaped by intra-actions of medical visualisation, digital infrastructures, and parental communities. In this sense, parental wellbeing is itself an emergent phenomenon: not an individual psychological state but the outcome of sociotechnical assemblages in which human and nonhuman entities intra-act to produce affective states.

In sum, the theoretical framework situates ultrasound and digital foetal images as material-discursive practices, which inseparably entangle medical, social, and technological dimensions. This perspective allows us to explore how parental wellbeing emerges in relation to the hybrid corporealization of the foetus—an analytical lens that informs our empirical investigation of mothers’ online narratives and the digital circulation of 3D/4D sonograms.

## 3 Methodology and data

This study explores the intertwined relationships between the transition to parenthood, parental wellbeing and processes of (de)corporealization as they emerge in online narratives surrounding three- and four-dimensional ultrasound technologies. Rather than aiming to reconstruct individual lived experiences or psychological states of expectant parents, the research focuses on digital environments to observe the production, circulation and negotiation of meanings, emotions, and bodily imaginaries in process. The study analyses online narratives not as transparent reflections of experience, but as constitutive sites for the material and discursive enactment of foetal bodies, parental subjectivities, and wellbeing.

The research adopts a qualitative approach informed by Multimodal Critical Discourse Analysis (MCDA) (Machin, 2013; van Leeuwen, 2013). MCDA integrates Critical Discourse Analysis with a systematic attention to multimodality, enabling the analysis of meaning production through the interplay of text, images, emojis, visual framing, and other semiotic resources that are characteristic of digital communication. This approach is particularly suited to the study of digital environments, where language, images, and visual-symbolic resources operate together in shaping shared meanings and affective responses.

The study is grounded in a socio-material and feminist STS epistemology (Haraway, 1996; Barad, 2007), according to which digital practices are understood as material-discursive practices. From this perspective, ultrasound images are not neutral representations of a pre-existing biological reality; rather, they are phenomena that emerge through sociotechnical assemblages involving medical devices, digital platforms, commercial actors, and parental communities. Consistent with Barad’s concept of *intra-action*, online narratives are analysed as practices through which foetal bodies and parental wellbeing are co-constituted.

This methodological choice responds directly to the research questions guiding the study: how digital visualisation and narration shape parental wellbeing and the foetal body, anticipating and reconfiguring the transition to parenthood. While interviews or ethnographic observations could offer valuable insights into embodied experiences, the present study intentionally focuses on publicly articulated narratives and visual practices, as these are the very sites where the phenomena of the *digital foetus* and *digital birth* take shape.

The empirical material consists of posts collected from two major UK-based forums dedicated to parenting. These forums were created for parents, but they are used primarily by young women seeking practical support and expert guidance on pregnancy and early childhood, as well as for sharp political debate and parenting advice. Both platforms are widely recognised as key digital spaces for exchanging advice, experiences, and emotions related to pregnancy and parenting. While registration is required to actively post content, the forums we analysed are publicly accessible for reading, making them a significant arena for the circulation of shared parental discourses.

To select the empirical material relevant to the aims of this research, searches were conducted on both online forums in sections explicitly dedicated to pregnancy, prenatal testing and experiences with ultrasound.

A targeted sampling strategy was adopted in order to identify discussion threads in which 3D/4D ultrasounds were explicitly mentioned, shared, commented on or evaluated. The data were collected through manual search and selection, rather than via automated scraping tools. The final dataset thus obtained consists of approximately 50 discussion threads, including text posts and, where available, embedded images or references to shared scans.

The posts selected in this way cover the period 2011–2024, encompassing the years in which 3D and 4D ultrasound technologies became increasingly available through private non-medical providers in the UK.

The analysis focused on the discursive and visual practices through which ultrasound images are interpreted, re-signified, and emotionally invested. The material was coded thematically using NVivo software. Coding was iterative and abductive, moving between empirical material and theoretical concepts, paying particular attention to:

- Narratives of parental emotions (e.g., reassurance, joy, anxiety, fear, disappointment).
- Practices of digital mediation (e.g., image editing, decoration with frames or emojis, transformation into memes).
- Discourses of bonding, anticipation, and wellbeing linked to the transition to parenthood.
- Aesthetic and moral evaluations of foetal images (e.g., references to beauty, resemblance, health, and gender).
- Linguistic and visual expressions that contribute to the objectification or personification of the foetus.

Rather than aiming for representativeness, the analysis seeks to identify recurring patterns, tensions, and ambivalences that characterise the construction of foetal bodies and parental wellbeing within these digital spaces.

The paper follows established ethical guidelines for qualitative research using online data. Ethical considerations were central to the research design. The study relies exclusively on publicly accessible forum posts, with no requirement for registration to read content. No interaction with forum users occurred, and all usernames and identifying details were anonymised. Quotations were selected and presented in ways that minimise traceability, in line with ethical guidelines for internet-mediated research. Ethical self-constraint motivates the authors’ decision not to include foetuses’ pictures in the presentation. Given the sensitivity of pregnancy-related experiences, particular care was taken to contextualise excerpts analytically rather than sensationally.

## 4 Online foetal body narratives as intra-action of parenthood transition

Following Barad’s theoretical framework (Barad, 2007), online narratives posted on analysed forums in relation to 3D and 4D ultrasound scans are understood as intra-actions—that is, as material-discursive practices involved both in the processes of the foetus’s corporealization and in the shaping of parental wellbeing. This approach made it possible to identify the main dimensions characterising these intra-actions.

From this perspective, digital narratives surrounding the foetal body are analysed as a *technology of parenthood transition*, emerging as sociotechnical assemblages in which medical, cultural, affective, and economic practices converge, while also remaining in tension with one another. The following sections present and discuss the main research findings in detail.

### 4.1 Sharing online foetus’ pictures as parental emotional training toward happy parenthood

Despite its questionable scientific validity, the *theory of ultrasound bonding*—the idea that foetal ultrasound constitutes a useful tool for fostering parental emotional attachment to the foetus—has acquired an appearance of credibility within medical literature and public discourse, to the extent that it is now invoked across a wide range of professional, political, and commercial domains (Taylor, 2008). Public communication on pregnancy health—promoted by both public and private medical institutions—emphasises the opportunity offered to parents to see and emotionally (re)cognise the future child prior to birth (Draper, 2002) and, moreover, to encourage fathers’ involvement in care responsibilities during the pre-birth period (Bestetti et al., 2022).

This intertwining of visual recognition and emotional engagement is particularly emphasised in relation to 3D and 4D ultrasound scans, strongly promoted by the private clinics that promise early encounters with the unborn child and transforming foetal attachment into a purchasable service.

In analysed online discourses, the pursuit for affective bonding—both for parents and for other family members— results one of the main motivations for undergoing a 3D or 4D scan, and to post and comment these images in digital forum contribute to reinforce this emotional practices.

I had a gender scan this pregnancy so got some 3D/4D pics from that and I’m having another one at 29 weeks and taking my daughter along so she feels involved (31/01/2018).

Here’s mine at nearly 26 weeks. Would definitely recommend the scans. I’m obsessed with looking at this photo now and imaging what he might look like when he arrives (3/4/2021).

Part of the reason for having one for the last two babies has been so the older ones can feel more bonded to baby. It’s one thing to talk about mummy’s tummy and to feel the odd kick, but to see baby’s face? That makes it real (16/10/2013).

I had one and I’m so glad I did. It was an incredible experience and I took both of our mums and my sisters along which gave them an opportunity to bond with the baby too (31/01/2018).

The 3D image is not merely an index of biological presence, but a performative event mediating the transformation of expectant parents into social parents. Sharing, visualising and commenting 3D ultrasound pictures generate emotional investment, enabling parents to project affection, resemblance, and identity onto the unborn child. The online narratives analysed illustrate how the affective and symbolic power of parental bonding is socially performed and (re)produced. In pregnancy forums, narratives surrounding ultrasound images shape emotional responses and perceptions of attachment; foetal images and the discourses around them contribute to making parental bonds and feelings tangible and real. In some cases, women describe the ultrasound session as a moment of realisation, a concrete awareness of the coming child. When three- dimensional images reveal the foetus’s face or body, the scan is narrated as a joyful encounter—a first meeting that transforms an abstract idea into a visible and emotionally charged presence.

Hi S., here is our little boy at 27 weeks. I am measuring big so am having growth scans every 2 weeks (am 35 weeks now) – you will love the 4D scan, that’s when it all became real for me (12/03/2017)

At first, I felt a bit like maybe we’d spoiled the surprise of seeing her little face in real life. But the more I looked at the pictures, the more I fell in love with them. It’s lovely looking at her chubby cheeks and pouty lips as she already looked like me! (25/01/2022).

Sara Ahmed, who in The Promise of Happiness (Ahmed, 2010) analyses technologies and objects as devices that align individuals within affective communities, offers a particularly relevant reflection for interpreting these practices. According to this perspective, the analysed digital narratives work as an apparatus aimed at normalising the emotions and feelings of parenthood transition. Through these practices, foetal visualisation becomes a site around which subjects orient their emotions and expectations, where affect and imagination converge into a framework of “happy parenthood.” As family photographic albums, the online forums, where the 3D scans are shared, construct parenthood as “happy object” (Ahmed, 2010), imposing normative expectations of parenthood, especially for mothers. They frame feelings and emotions of good mothers and fathers, easing, or rather hiding, the anxieties, uncertainties and unhappiness associated with the transition to parenthood.

### 4.2 Online narrative on “baby watching” as practices of objectification of the foetus body

In online pregnancy forums, it is common for mothers to engage in the exchange of information regarding the quality of ultrasound centres, the costs, and the appropriate timing for undergoing an ultrasound examination.

I really want a 3 or 4D scan but was just wondering how many weeks people recommend having it done? I’m 14 weeks Now and I am thinking of having one at around 17 weeks or around 28?

R: all my scans have been in 4d, which means the images are moving (private health care here in dubai 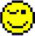). However we have a proper “baby watching” session at 27 weeks which i was told was the best time. Any later and the baby is too big and you won’t get the movements. Any earlier then it is really difficult to see clearly (02/09/2011).

In this last post, we focus on the expression “baby watching,” frequently used in online pregnancy forums to describe the experience of foetal ultrasound. This term marks a significant semantic and ontological shift in the cultural and social understanding of prenatal imaging. Whereas expressions such as “sneak peek” still evoke curiosity or anticipation—suggesting a glimpse into an ongoing biological process—“baby watching” explicitly describes ultrasound as the act of watching a baby, that is, of viewing a separate, already individuated body.

This linguistic practice contributes to the foetus being no longer imagined as part of the maternal body but as an autonomous subject, an object of vision that exists independently from the woman who carries it. Within this framing, the ultrasound session becomes an event of observation rather than of embodied experience—a moment in which the foetus is constructed as a visible, knowable, and externalised entity.

This semantic shift reflects what feminist scholars have long identified as the objectification of the foetus through visual technologies (Petchesky, 1987; Duden, 1994; Franklin, 1991). The ability to “see inside” the maternal body paradoxically contributes to the invisibility of the woman herself: her corporeality becomes transparent, serving merely as a medium for visualising the foetus. As the womb turns into a window and the foetus into a show, the technologically mediated gaze displaces pregnant woman’s embodied experience — privileging visibility and detachment over sensation and connection.

In this sense, “baby watching” encapsulates the epistemic and cultural transformation induced by sonographic practices: it converts an intimate, bodily process into a scene of observation, where the foetus becomes a separate object of affection, diagnosis, and display. What is being watched, ultimately, is a technologically produced representation of an “othered” body, detached from the woman’s own corporeal experience of it.

However, in the mothers’ narratives, this process of objectifying the foetal body is constantly negotiated and remains in tension with the (sometimes weak) awareness that the foetus is not yet an autonomous, fully formed baby’s body, but is rendered by the working of the ultrasound technologies themselves. The objectification of the foetus is thus never complete; it unfolds through a series of interactions and interpretive acts in online discussions. Users exchange advice about the “best places to watch the baby”— that is, the ultrasound centres reputed to offer high-quality images — and about the ideal “foetal season” for observation, meaning the gestational weeks that provide the most satisfying visual results. In these conversations, they often describe early scans as disappointing, because foetuses do not yet display recognisable baby-like features, while late scans are frustrating because only partial body sections can be seen.

Moreover, not only discourse but also material constraints contribute to the objectification of the foetus. The maternal body, made only partially “transparent” by the ultrasound technology, still resists complete erasure. Physical elements such as the position of the placenta, uterine walls, or amniotic fluid density may obstruct the visual field, interfering with the production of clear 3D images. The anterior placement of the placenta, for example—a condition generally considered physiological and not affecting foetal health—often results in distorted or blurred sonographic images of the foetal face or body. A mix of irritation and disappointment emerge in comments about these visual interruptions, which act as material reminders of the foetus’s embeddedness within the maternal body, are with. Mothers express frustration that their bodies, rather than functioning as transparent media of revelation, seem instead to obstruct access to the visual truth of the foetus.

Thus, even as the digital and medical apparatuses strive to disembody the foetus—to render it as an autonomous visual object—the maternal body continues to assert its presence, materially and symbolically. In these online accounts, the persistence of the maternal body is not perceived as connection between mother and foetus but as a source of interference, a barrier to the full visual and emotional realisation of the “baby.” In this tension between visibility and opacity, autonomy and embodiment, the very process of foetal objectification becomes apparent: it is never a simple technological act of seeing, but a socially mediated and materially constrained practice through which social meanings, affective expectations, and bodily realities continuously intersect.

My first son I had an anterior placenta. I had a 4D scan at 25 weeks and 28 weeks. I’ll attach the 28-week ones first, then the ones at 25 weeks. They had more problems getting good shots this time and my placenta is on my right-hand side – he therefore basically had the placenta wrapped round his face (18/5/2017).

Another process contributes to the objectifying the foetal body: it is the sharing of ultrasound images as pregnancy selfies. Once released from the clinical context, these images circulate in digital ecosystems where they are displayed, commented upon, and creatively reworked. Young expectant mothers—that, according to the studies on online parenting forums, are the primary users of these platforms —engage in these practices in ways that mirror the selfie culture of social media. These women are accustomed to sharing snapshots of everyday life; thus, posting ultrasound scans becomes an extension of this digital habit, a way to involve friends, family, and even strangers in their pregnancy journey. These shared images allow parents to “introduce” their babies to the world well before birth, anchoring the unborn child’s presence in social life (Agodi et al., 2023).

As with selfies, these practices are not limited to uploading a neutral image. The posts’ authors *decontextualize, annotate and decorate* ultrasound images with digital embellishments such as frames, pink or blue hearts, ribbons, and sometimes transform them into memes. Paradoxically, in these “pregnancy selfies” the *maternal body* is *absent*. Made transparent by the ultrasound apparatus (Van Dijck, 2005), the mother herself is removed from these visual and textual narrative. Within the textual corpora too, explicit references to the mother’s body are almost completely absent, appearing only once across some threads. This invisibility reinforces the foetus as a discrete, autonomous subject. In this sense, the foetus scan, although transposing the pregnancy experience within the frame of the selfie culture, is representing the foetus as the object of the selfie itself.

Absolutely loved having these scans! Such an amazing experience watching what the little one is up to whilst feeling them moving about at the same time. Worth every penny xx (12/03/2017).

Consistently with this embeddedness in the selfie culture, the images carry implicit messages about *gender and beauty*. Comments about appearance and resemblance accompany (foetal) selfies —such as:

Sadly my maternal grandmothers family conk was very visible in the scan and the moment she was born it was what we noticed first. Luckily it’s not as noticeable as she’s gotten older 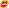 (28/04/2023).

I had a scan last night and my little girl is bit how I imagined her. She looks the spit of my husbands family and I couldn’t help but cry. Some of the kids in his family are ugly and our baby looks like his mum 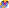. Has anyone ever been really worried about having a ugly baby? Does any new parent actually look at their child and think ‘oh you are so ugly’? 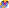 I feel so horrible saying this but I’m honestly worried. I have a daughter from a previous relationship and she is beautiful but always has been from day she was born even her 4d scan she had a cute button nose and looked just like her scan she was smiling in it. The new baby literally had this miserable grumpy awful face just like my mother-in-law 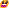 (25/01/2020).

These remarks reflect how foetal images feed into *normative beauty standards* and *gender stereotyped expectations*. Parents project notions of attractiveness and familial resemblance onto the unborn, prefiguring their child’s social identity. This aligns with broader dynamics observed in media studies: aesthetic norms and gendered appearance ideals are pervasive and shape expectations from the earliest moments of life.

One recurrent theme is the eagerness to see the baby before the birth and to know the sex as early as possible, often before clinical confirmation is reliable. Parents post 3D images asking peers to “guess,” turning the foetus into an object of collective interpretation. Some parents circulated images of their foetuses and asked peers to “help” identify features of the foetus, reinforcing the idea that the digital foetus is socially co-constructed through online guessing games.

### 4.3 Misalignment between socially constructed happy parenthood, objectified foetal images, and lived viewing experiences

Although most women describe their ultrasound experiences as amazing and are willing to share images of their foetuses on online forums, these encounters do not produce the always reassuring and emotionally rewarding visualisation promised by contemporary ultrasound culture. As shown in the previous sections, 3D and 4D scans social framing depicts them as technologies capable of supporting bonding, happiness, and emotional certainty during pregnancy. However, empirical narratives reveal that this promise is fragile and unevenly fulfilled.

Ultrasound technology opens a “window to the womb”, enabling access to a world otherwise invisible to the human eye. This possibility generates excitement and curiosity, but also anxiety, discomfort, and ambivalence. The experience is frequently referred to as a “sneak peek,” an expression that conveys the sensation of accessing a vision that is at once desirable and transgressive—premature, forbidden, or excessive. In this sense, the visualisation of the foetus is not a neutral act of seeing, but an emotionally charged encounter shaped by anticipation, norms, and expectations.

The “pre-view” of the baby is therefore imbued with ambivalent emotions. On the one hand, seeing the foetus “in advance” is expected to intensify joy and attachment; on the other, it disrupts the temporal structure of pregnancy, traditionally oriented around anticipation and waiting. Several women describe the act of viewing the foetus as a form of trespassing—an intrusion into a space that should remain private until birth. Online forums play a crucial role in mediating this tension: mothers use these spaces to seek advice, exchange experiences, and emotionally prepare for what they often describe as a “stolen vision,” a technologically enabled gaze that exceeds ordinary human perception. This emotional training emerges clearly in early forum posts, where expectant mothers articulate conflicting reactions to 3D imaging:

It was moving and very emotional. But the 3D was freaky. Not just because of the particular way the picture look but it was like something you’re not supposed to see. Like you have peeked at a secret and now you cannot un-see it. Hard to describe but a very strange experience (17/07/2013).

I had a scan last week at 16 weeks and in order to completely confirm the gender the sonographer switched to 3D imaging. I turned my head away and thank god I did because she then did a quick sweep over the rest of him. I would have been mortified if I’d seen anything. The first time I want to see my babies face and beautiful features is when he is handed to me after the birth. I think it is amazing what scans can show but this whole 3D/4D thing just isn’t for me. I think it takes away some of the magic (16/10/2013).

In preparation for the transition to the foetal world, mothers engage in discourse and the exchange of information on forums to understand in advance of the visual depictions by ultrasound scans, the emotions these may evoke, and the circumstances under which this experience may be deemed valuable (and consequently the financial investment it requires).

Hello ladies, I’m currently 24 weeks exactly and since the beginning of my pregnancy I’ve been debating whether to have a 3D/4D scan. Some people have said they would highly recommend having one and go on with how amazing they are. Then on the other hand some people have said that it’s just more of a way to waste money and don’t see much point in it, as you still get to see your baby when you have your 12 week scan and 20 week scan. What are your opinions on the 3D/4D scan and has anyone gone for one? And if so what was the experience like? (31/01/2018).

I’ve booked a 3d/4d scan for Wednesday and I am SUPER excited I’m going to be 30 weeks and 3 days! What’s everyone’s opinions on them and does anyone have any 3d/4d scans they could show from around 30 weeks (03/04/2021).

Consequently, the misalignment between the objectified perspective of the foetus and the actual viewing experience can engender feelings of frustration, and occasionally, even genuine anxiety; or, at best, it is evaluated as a waste of money.

Many parents articulate feelings of discomfort or even horror when confronted with the 3D/4D images. For some, the foetus appears “alien-like,” “creepy,” or disturbingly distorted. Some users confessed:

We had one and absolutely hated it. I found it very distressing, I couldn’t shake that the images looked like photos of stillborn babies and it exacerbated my already sky high anxiety. Not only that, but her face was squished right against my placenta so she looked exceptionally ugly and I spent a long time really panicked that she would be ugly!!! Hopefully you will have a lot more success than we did and it is good to look at examples to know what to expect! Xx (03/04/2021).

I’m 34 weeks pregnant and have just got back from a routine growth scan […]. The sonographer printed out a 3d scan of the face and I am totally freaked out by it. The picture just looks odd to me. It has massive lips and a big nose which neither dh or I have. I’m not really sure how to explain this, but I just can't equate the picture with the baby I have inside me. I wish I’d never seen the it. I actually feel disturbed looking at it (although compelled to do so).

R: 3D scan was horrific! She had a massive forehead, huge lips and what looked like a great big plasticine nose. She came out looking like a squashed baby and has grown into a pretty 6 year old. Take the pic with a pinch of salt and don’t forget that they are distorted by the water/placenta so most aren’t a true representation x (17/07/2013).

Such testimonies illustrate the *uncanny status* of the digital foetus. While intended to provide reassurance, the images may instead destabilise maternal wellbeing, generating doubts about health, beauty, or normality and finally about happiness. The foetus becomes both intimate and estranged, a paradox that speaks to the performative power of these images. The ambivalence is growing when sonographers introduce unsolicited medical comments during private scans. For instance:

I was happily pregnant until I went to do a 3d ultrasound at 29 weeks … the sonographer said that her femurs looked a bit short … Even though it was lovely to see my baby I wish I’d never gone to do these scans … I just want to cry from all this stress (22/05/2024).

I had a freebie 3d/4d with my last and hated it. It’s creepy and baby looks nothing like how they do once out. I regretted it quite a bit. I’m not doing it this time (25/01/2022).

At the same time, the online communities of mothers implement narrative practices, both discursive and iconographic, aimed at exorcising the fears that foetal images sometimes generate, and at supporting other women in their fears.

I find 3D scans terrifying in general. We got a free one as part of our gender scan, but we hadn’t realised that when we booked. We’d both spoken before about how freaky we find them and how we didn’t want one, and then suddenly popped up our darling baby girl, looking like a combination of a melted candle and The Scream painting by Edvard Munch. Here she is, to haunt your nightmares… We both cracked up when we saw her. I’m saving it for her 18th birthday (27/09/2018).

## 5 Reconfiguring timescapes of parenthood transition between platform pregnancy and digital birth

This article has explored how online narratives surrounding three- and four-dimensional ultrasound scans contribute to the social (de)construction of foetal and maternal bodies, and how these processes intersect with parental wellbeing during the transition to parenthood. By analysing posts shared on two UK parenting forums through a Multimodal Critical Discourse Analysis, the study has shown how foetal imaging practices extend well beyond their clinical function, while becoming embedded in digital, affective, and consumer cultures of pregnancy. Through these practices, parental wellbeing emerges as a relational and ambivalent process—constantly negotiated between reassurance and anxiety, connection and detachment, visibility and vulnerability. From a sociological perspective, these findings contribute to ongoing debates on the social construction of bodies, the mediation of care and intimacy, and the role of digital infrastructures in shaping contemporary forms of subjectivity and kinship.

The analysis reveals that online discussions around 3D and 4D ultrasound images perform a crucial role in mediating emotional experiences during pregnancy. Parents’ exchanges in forums and social media act as infrastructures of emotional regulation, where uncertainty and fear are collectively processed. In these digital environments, individual distress—often caused by the misalignment between the objectified image of the foetus shaped by cultural expectations and the personal visual experience of ultrasound— evolves into shared narratives of resilience and reassurance. Through collective commentary, peer validation, and empathy, online communities help restore emotional balance and normalise ambivalent feelings. These interactions illustrate how wellbeing, rather than being a purely individual psychological state, is co-produced within sociotechnical assemblages that bring together human and non-human actors—parents, sonographers, sonographic machines, public health institutions and private clinics, platforms, images, and others users.

In this sense, online discursive practices around three- and four-dimensional ultrasound can be interpreted as technologies of transition to parenthood. This process exemplifies what we define as *platform pregnancy*: a mode of experiencing and narrating pregnancy that is deeply shaped by the affordances, temporalities, and affective economies of digital platforms. Through digital visualisation and circulation, the process of becoming a parent no longer unfolds solely through embodied, sensorial, and temporally extended experiences, but through digitally mediated acts of seeing, posting, and narrating. The sharing of ultrasound images, the collection of comments and emojis, and the incorporation of the foetus into online profiles or digital decorations all contribute to an anticipatory socialisation of the unborn. These practices collapse the distinction between the expectant and the actual, between pregnancy and parenthood. Wellbeing is thus negotiated within complex assemblages of affect and accountability: the desire to share joy coexists with the fear of judgement, and reassurance is constantly sought through online validation. Parental subjectivity emerges through this oscillation between exposure and belonging, visibility and vulnerability.

Online exchanges also constitute a digitization of kinship, where affective bonds are enacted and affirmed through digital traces. Through these interactions, the foetus becomes part of a visual and relational economy of recognition that includes not only the parents but also their social networks. The unborn child is thus inscribed into a field of relational expectations even before its physical appearance, transforming the affective architecture of family life.

Building on Barad’s (2007) concept of apparatuses as material-discursive practices, the analysis suggests that digital platforms intra-act with sonographic technologies and parental discourses, producing the foetus as a digital object endowed with visibility, sociality, and affective presence. The ultrasound image becomes a site of multiple entanglements—between medical expertise and lay interpretation, emotional projection and technological mediation, intimacy and publicity. Parents’ acts of sharing and commenting confer symbolic and social life upon the unborn, situating it within what Haraway (1996) called processes of *corporealization*—the making of bodies through networks of material and discursive practices. Through its digital circulation, the foetus is not only made visible but also real, acquiring cultural and emotional substance.

Our findings extend feminist critiques of the *public foetus* (Duden, 1994; Taylor, 2005) by documenting the emergence of the *digital foetus*: a hybrid and distributed entity that takes shape within digital infrastructures of visibility and affect. This shift has important consequences for parental wellbeing, as it relocates and apparently move to the socially exposed sphere the emotional labour, responsibility, and recognition of parenting in a phase of life that precedes embodied interaction, amplifying both attachment and social vulnerability. Unlike the public foetus—whose visibility was primarily constructed through medical and political representations—the digital foetus is actively co-produced by parents themselves, who upload, edit, and circulate sonographic images across social media and online forums. These practices generate what we called the *digital birth*: the entry of the foetus into social life before biological birth occurs. In this sense, digital imaging and online sharing function as technologies of transition to parenthood, overlapping temporalities and transforming the thresholds that once separated pregnancy, birth, and the recognition of the newborn as a social subject.

This process entails profound temporal transformations. Online narratives show the acceleration in the experience of pregnancy within digital temporalities, where anticipation and visualisation become central affective modes. The possibility of “seeing” the unborn child through 3D or 4D imaging, combined with the instantaneous sharing and commenting afforded by digital platforms, produces a temporal shift: the foetus is experienced as already present, already visible, already part of the social world. The *digital birth* therefore precedes and anticipates biological birth, reconfiguring the temporalities of parenthood itself.

Drawing on the notion of *digital timescapes* (Picardi, 2025), we can interpret these transformations as manifestations of the temporal reorganisation produced by digital infrastructures. As Adam (1998) argued, timescapes are the lived, relational, and heterogeneous temporalities through which social life unfolds. In the digital age, logics of immediacy, simultaneity, and visibility, embedded in technological systems, are increasingly shaping these temporalities. The digital timescape of reproduction reconfigures the temporal order of pregnancy: it anticipates birth, compresses waiting, and intensifies the affective engagement—or detachment—with the unborn. Within this altered temporal horizon, the *digital foetus* becomes both a temporal and affective figure—a presence that embodies the acceleration and synchronisation characteristics of digital life, through which digital infrastructures actively reshape the experience and governance of reproductive time.

These dynamics have profound implications for the understanding of parental wellbeing. The *platform pregnancy* entails a reconfiguration of kinship and family narratives. Its ambivalence is central: on the one hand, digital imaging and online sharing foster intimacy, joy, and anticipation, reinforcing the parental bond and embedding it in supportive communities; on the other, they heighten anxiety, amplify aesthetic and medical concerns, and blur the embodied temporality of pregnancy. The immediacy of digital feedback loops contrasts with the slow, rhythmic unfolding of gestation, potentially erasing its sensory and corporeal dimensions. This tension captures the paradox of wellbeing in the digital age: a state continually mediated by technologies that promise control and reassurance but also generate new forms of exposure and affective instability.

In conclusion, our analysis shows that three- and four-dimensional ultrasound technologies, intertwined with digital platforms and online communities, are transforming the temporal, emotional, and social dimensions of the transition to parenthood. The *digital foetus* emerges as both a product and a producer of these *digital timescapes*—an entity through which the rhythms of pregnancy and parenthood are reorganised, and the boundaries between life stages are redefined. Understanding parental wellbeing in this context requires recognising how it is entangled with digital infrastructures, aesthetic norms, and collective emotions. Becoming a parent today means navigating these altered temporalities, negotiating between embodied experiences and digital representations, between the slow temporality of gestation and the instantaneous visibility of the digital. In this convergence of biological and technological time, the ethics and emotions of reproduction and parenthood are reconfigured, revealing how visual technology in medicine embedded in the digital world reshapes not only what it means to see life before birth, but also how we inhabit and imagine the time of becoming. Attending to these digital timescapes of reproduction is therefore crucial for understanding how bodies, emotions, and social relations are being reconfigured in contemporary societies, and for critically interrogating the promises of wellbeing embedded in platform-mediated forms of care and intimacy.

## Data Availability

The datasets presented in this article are not readily available because the empirical basis of the research is provided by the ethnographic diaries kept during the webethnography. These ethnographic diaries are kept by the authors and are not accessible. Requests to access the datasets should be directed to ilenia.picardi@unina.it.
